# Baths and Bathing

**Published:** 1881-08

**Authors:** 


### BATHS AND BATHING.

[From Dr. Hall's Family Doctor Book.]

NO ROOM in which a bath is taken should be of a lower temperature than seventy-five degrees F., so that there will be no unpleasant sensation of coldness when the body emerges from the water.

For ordinary purposes of cleanliness the bath should be of warm water, with soap rubbed well in with the hand or sponge, reaching every part of the body possible, rinsing off afterward, so that there shall be no soap left on the skin; in wiping dry with a towel, friction should not be spared, laying the towel flat on the hand, keeping the mouth closed, then rub with a will nearly as hard as the hand can press, then for a minute or two more rub in the same way all over with the hands; this tends to soothe and soften the skin and impart a pleasant warmth to the surface. In taking this warm cleansing bath, the water should indicate ninety five degrees F.; fifty for a cold bath; seventy-five tepid; a hundred and five hot. In taking a tepid or

COLD BATH

the head should be wet with cold water, or forehead and face well washed with cold water, before getting into the bath tub. If the feet are cold, they should be soaked in hot water, half leg deep, until completely warm, before getting into the bath. These baths should be taken rapidly, the hand kept gently rubbing the limbs and body all the time, so as to keep up the circulation and thus prevent a feeling of chilliness, and, for the same reason, as soon as the body is out of the water it should be promptly and actively rubbed with a towel until perfectly dry, for if a feeling of chill runs over it, a great deal more harm has been done than good, by the whole process; in addition, dress quickly, and if not perfectly comfortable as to warmth, take a brisk walk immediately, or chop or saw wood, or do some other thing which will restore the circulation; if at night, or the weather is unsuitable, either go to a good fire, or into the kitchen, or to bed and wrap up, or dance vigorously until a sensation of comfortable warmth has been brought about, for persons have taken a chill in the bath which has resulted in death. The precaution should always be taken, in all forms of bathing, to stand on a piece of woolen carpet or mat in emerging from a bath, because it is essentially important that the feet should be warm; for coming in contact with the cold floor or oil-cloth a chill may be sent through the whole body. A gentleman in the advanced stage of consumption attributed his disease to standing on a piece of zinc on the bath-room floor, after taking his bath and while rubbing himself dry.

### TIME FOR BATHING.

Persons in vigorous health, near the sea, or river or lake, may safely walk half a mile before breakfast, jump in over head, swim around for five or ten minutes ; dress quickly, walk rapidly home and take breakfast, and be all the better for it in summer time. But for all ordinary purposes, a bath should be taken about ten or eleven o'clock in the forenoon ; having taken breakfast three hours earlier, for the human system is more vigorous at that time of day than at any other hour of the twenty-four, as it is at its lowest ebb about five o'clock in the morning ; hence it is that more persons die at that hour than at any other, under ordinary circumstances.

A bath can do no good, but will always do harm if a person is

### TIRED.

Nor should any kind of a bath be taken sooner than three hours after a regular meal, nor later than an hour or two before eating. Many persons have been found dead in the bath-room from taking a bath soon after a hearty dinner, even a warm bath.

### BATH FOR FEVER.

If a person goes into a cold bath to cool off a fever, a hurtful and dangerous shock and chill may take place ; it is safer and far better, far more comfortable, and more efficient in cooling off the body, to have the water at eighty-five, bathe for a while, then gradually reduce it, but by no means to the extent of causing an uncomfortable feeling of coldness. It is safer to bathe in warm water to cool off a fever of the whole body, throwing the water upon it, running off, and thus allowing the evaporation to carry off the surplus heat.

In all forms of bathing, hot, cold, or tepid, a damp towel or wetted cap should be worn on the head, or if the hair is short, it should be wetted several times, the most effective way of doing which is to douse the whole head and ears under water, the object being to prevent an excess of blood from going to the head ; fatal apoplexy has resulted in many cases from failing to take this precaution of keeping the whole head wet or cold in all forms of bathing. The article used for wiping off the body after a bath should be soft.

If a person takes a bath in a frail or feeble condition of the system the whole body should be invested in a sheet the moment of emerging from the water and rubbed dry with it; this prevents chilliness. Then the whole surface should be rubbed with the hands, all over the body as far as can be reached in every direction, mouth closed, bearing on with the hands as hard as convenient; this aids in promoting the circulation, in expanding the lungs, and softening and warming the skin. If a person is feeble, then this hand rubbing should be performed by an attendant; in either case it should be done with rapidity and energy, and completed within three minutes if there is any tendency to chilliness, for if a single chill runs over the body, or along the back during or soon after the bathing, more harm has been done than good, hence any feeling of chilliness should be guarded against with great care. After dressing rapidly, the invalid should go to the fire, or into the sunshine, while those that are able should take a brisk and extended walk, or engage in some form of vigorous exercise involving muscular activities in the open air, to invigorate the circulation and bring it to a healthful glow to the very extremities of the system. Sometimes in feeble persons it is best to go to bed, wrap up warm, and perhaps go to sleep, for to them rest from the fatigue of the bath is of more importance than exercise, the warmth of the bed keeping up the circulation better than motion of the limbs. But in all cases of bathing, if, after a couple of hours, there is a sense of weakness or fatigue or tiredness, an indisposition to move around, then the bath has been an injury.

FOOT BATH.

It is better to have a wooden pail, not very broad, so that it need not require a great deal of hot water when a hot foot bath is taken, for when such a one is necessary it is important to add hot water from time to time, that it may be at least as hot when the feet are taken out as when put in. The vessel should be deep enough to allow the water to come up near the knees.

TO TAKE A SWEAT.

Get into a sitz-bath, with the feet in a hot bath, all as warm as can be well borne, cover the whole body, and add hot water enough from time to time as the other cools ; keep the head well wet with cold water all the time ; after the perspiration has continued long enough, take quickly a half-bath or dripping sheet, at not lower than eighty degrees for an instant only, and then go to bed at once. The favorite hydropathic method of curing a bad cold recently taken by a healthy person is to take a profuse sweat, go instantly to a pack of eighty degrees, followed by a dripping sheet, go to bed, and for two or three days eat sparingly of coarse breads, fruits and vegetables.

### WHEN TO AVOID BATHS.

When followed invariably by headache, chilliness, or other discomfort. When a decided glow does not follow the operation, although there may be no chilliness.

Avoid cold baths when the skin is moist, or the pores of the skin are open, and the sudden closing of them may cause chilliness or internal local congestions, more or less dangerous.

Not within an hour of eating a regular meal, or at least two hours after. Not when tired or debilitated, as, for example, after a long walk or ride, or at the close of an over-busy day. Not when hungry.

### SEA BATHING.

It is not denied that persons are benefited at times by going to the seashore; but the fewest number of invalids are there, in reference to whom it can be said with certainty that any benefit derived was the result of sea bathing; the simple change of air, of scene, of cookery, of associations and habits, having advantages which, in many cases, are wrongfully attributed to sea bathing.

### TEMPERATURE OF BATHS.

To remember easily, it is sufficient to say that—

A cold bath is fifty degrees or under.

A tepid bath about seventy-five.

A warm bath about one hundred.

A hot bath about a hundred and ten.

A vapor bath about a hundred and fifteen.

A cold bath is tonic.

A tepid bath is cleansing and calming.

A hot bath relaxes the muscles and excites the circulation.

---

William Herndon, the law partner at one time of Abraham Lincoln, and once in affluent circumstances, is now a pauper in Springfield, Ill. He was brilliant of speech and profound in the judgment of his profession, but being of strong appetite and free social habits, he became addicted to drink, sank into degradation, and is now a public charge. Tom Marshall, once famous for his learning, his vivid imagination and brilliant oratory, was also a victim of rum. And how may others of like genius at the bar, in the counting-room, the workshop and the field have fallen by the same influence?

